# Sterility Assurance Across-Sectors—New Paradigms and Tools

**DOI:** 10.3389/fmedt.2021.622710

**Published:** 2021-08-09

**Authors:** Byron J. Lambert, Joyce M. Hansen, Trabue D. Bryans, Stan Lam

**Affiliations:** ^1^Abbott's Assurance of Sterility Task Force, Divisional Engineering, Abbott Vascular, Temecula, CA, United States; ^2^J&J Microbiological Quality & Sterility Assurance, Johnson & Johnson, Raritan, NJ, United States; ^3^BryKor, LLC, Marietta, GA, United States; ^4^Process and Technology Development, Stryker Neurovascular, Fremont, CA, United States

**Keywords:** sterilization, aseptic processing, sterility assurance, sterility assurance level, probability of a non-sterile unit, medical devices, combination products, sterilization standards

## Abstract

The sterility assurance community is facing significant challenges. A relatively recent challenge is the pressure on manufacturing supply chains resulting from the limited availability of capacity for terminal sterilization of healthcare products. The current challenge is finding solutions for innovative new products, especially biologics and combination products, that offer great promise for patients around the world. This challenge will become more prevalent in the future as products advance. This article frames new paradigms and tools being developed to address these challenges. Foundational principles and current realities from each sector are reviewed so that sterility assurance professionals have a solid base from which to build strategies.

## Introduction

Sterility assurance for the healthcare industry spans many sectors: medical devices, pharmaceuticals, biopharmaceuticals, sterile barrier packaging systems, biologics/tissues, and healthcare facilities. These sectors utilize mechanisms to control the microbiological quality through aseptic processing of pharmaceuticals, biopharmaceuticals or combination products; terminal sterilization of packaging, components or final finished products; cleaning, disinfection, and sterilization of products and tools in healthcare facilities and reprocessing. Each of these sectors is regulated and the microbiological quality strategy selected is multi-dimensional. This complexity includes selection of a sterility assurance approach; developing a manufacturing operational strategy including microbiological controls; developing a validation strategy including implementation and validation; routine control of the process and product release; and sterile product distribution and acting on quality feedback. Sterility assurance professionals in each sector are required to manage this complexity. The first part of this article will review sterility assurance standards and current realities embedded into each industry sector to provide the foundation for innovative solutions. This is followed by discussion of new paradigms and tools that build on this foundation to solve current industry challenges.

There are major challenges facing sterility assurance professionals that are adding complexity and driving cross-sector awareness and collaboration. The first challenge focuses on the terminal sterilization industry and is driven by environmental and capacity pressures on the contract sterilization market, specifically, on ethylene oxide (EO) sterilization and gamma radiation (gamma) sterilization.^1^ These challenges forced the industry to understand capacity, optimize sterilization processes, qualify additional sterilization sites and/or vendors for business continuity planning, and/or look at modalities not currently widely available at contract sterilizers (e.g., dry heat, moist heat, alternative gases or X-ray). Each of these can drive significant resources and complexity to implement a different strategy and may take significant periods of time. Sterility assurance professionals provide great value in addressing these challenges through understanding the unique aspects of each sterilization technology along with its impact on product functionality, and through optimizing the validation strategies.

Another major challenge facing sterility assurance professionals is the growing amount of combination products and biopharmaceutical products that are not easily sterilized with traditional approaches. Combination products are defined by ISO^2^ as

Entity presented as a single health care product that physically, chemically, or otherwise brings together or mixes items regulated under separate legislation;
-Note 1 to entry: The entity could be a combination of medical device and medicinal product or biopharmaceutical product.

The discussion of sterility assurance innovation applies to product per this definition that includes any combination of device, pharmaceutical and biopharmaceuticals. The combination of medical device and medicinal product or biopharmaceutical product adds sterility assurance complexity because the different items combined may not have the same material compatibility with traditional sterilization processes. This complexity is compounded if the medical device also includes active electronics or bioresorbable materials, both of which have material compatibility challenges with the majority of the potential sterilization options. The term “sensitive combination product” can be used to capture this product combination with added sterility assurance complexity. Another aspect of this challenge is developing new regulatory paradigms to support high quality and high capacity aseptic processing. There is not an alignment between traditional regulatory paradigms and best aseptic processing technology (1, 2) that provides minimal risk to the patient to progress the aseptic processing sector.

Sterility assurance professionals often need a broad understanding of the different industry sectors to find optimal sterility assurance solutions; a resource toward this end was recently published (3). Finding solutions often requires new cross-sector paradigms for sterility assurance professionals and new tools for systematic analysis of the complexity. The second part of this article focuses on some recent new sterility assurance paradigms and tools to help sterility assurance professionals address this complexity.

## Sterility Assurance Standards and Current Realities

To provide a clear picture of the foundation upon which innovative solutions will be built in addressing the challenges facing the industry, this section surveys the different sterility assurance sectors and lists the robust ISO standards, Pharmacopeia requirements and other regulatory frameworks that are part of the sterility assurance toolbox. A high-level summary of how key sterility assurance principles from these standards are put into practice—our current reality—is also provided.

### Designating Medical Devices as Sterile

This section applies to all approaches to developing sterile product. We begin with standards that provide the regulatory definition of “STERILE” and an important summary of how the sterility assurance principles surrounding these standards have been applied. Both terminal sterilization and aseptic processing regulatory definitions are provided. Understanding this perspective and the new guidance recently provided to the industry in ISO/TS 19930 is foundational for developing innovative solutions to sterility assurance challenges facing the industry. ISO/TS 19930, *Guidance on aspects of a risk-based approach to assuring sterility of terminally sterilized, single-use health care product that is unable to withstand processing to achieve maximally a sterility assurance level of 10*^−6^, is a major recent step in bridging understanding between terminal sterilization and aseptic processing sectors and framing innovation for both.

#### Sterility Assurance Principles and Current Realities

At the highest level, product can be designated as sterile *via* exposure to terminal sterilization processing^3^ or manufactured using aseptic processing.^4^ In practice, EN 556-1 and EN 556-2 are the gold standards in the industry for designation of medical devices as “STERILE.” Terminal sterilization of the product is conducted after the product is sealed in its sterile barrier packaging system. Aseptic processing is the filtration sterilization and/or assembly of sterile components into a sterile barrier packaging system using controls to prevent the addition of microorganisms through the manufacturing process of the final product. Both of these paths provide sterile product, but they are not treated as equivalent paths. Terminal sterilization is preferred from a patient risk perspective. The preference is seen in the aseptic processing introduction of EN 556-2.

Medical devices designated as ‘STERILE' are prepared using appropriate validation methods. Whenever possible, sterile medical devices are terminally sterilized using a properly validated and controlled sterilization process (see EN 556-1, EN ISO 11135, EN ISO 11137-1, EN ISO 14160…). When medical devices are intended to be sterile but cannot be terminally sterilized, aseptic processing is the method of manufacture (see EN ISO 13408-1).

Aseptic processing necessitates that either:

- The entire product is sterilized and then introduced into a sterilized package; or
- Components of the product are sterilized before being processed/assembled, and packaged into a sterilized container.

The role of terminal sterilization according to the ISO standards for terminal sterilization is highlighted in the first requirement of EN 556-2:

“For an aseptically processed medical device, the following shall apply:

- the manufacturing environment in which the aseptic process is conducted is specified and records demonstrating compliance with the specification throughout the conduct of the process are prepared and maintained;
- the process employed to sterilize the product, components, equipment, and packaging are validated and routinely controlled in compliance with EN ISO 11135, EN ISO 11137-1, EN ISO 14160, EN ISO 13408-2, ISO 17665-1… as applicable.”

It is difficult to maintain the exclusion of all microorganisms from a manufacturing/packaging area. Hence, the preference is to utilize a validated terminal sterilization process as this is an active mechanism to destroy any microorganisms that might be on the product following assembly.

As discussed previously, active pharmaceutical ingredients and/or combination products can be adversely impacted by traditional terminal sterilization processes, and therefore terminal sterilization might not be the process selected to ensure a sterile product. However, a shift in the selection of sterility assurance levels >10^−6^, e.g., 10^−4^, per ISO/TS 19930 and using bioburden-based validation methods may open the door for terminal sterilization of traditionally aseptically processed product if bioburden is low in number, low in resistance and well-controlled.

This high-level view gets to the heart of putting the challenges facing the sterility assurance community into perspective. For the first challenge, the question to ask is, “Why is it difficult to change to alternative modalities (e.g., dry heat, moist heat, alternative gases, electron beam or X-ray) and/or to add capacity?” The answer is that product functionality constraints drive the selection of the terminal sterilization technology, and other terminal sterilization modalities may not provide the product attributes desired. In addition, many alternative modalities are either not available with the capacity (i.e., volume) necessary to process the high volume utilized by the healthcare industry, and/or alternatives modalities (e.g., alternative gases) may not have been proven to scale up for products in their sterile barrier system. The manufacture of high-volume medical devices is not possible without terminal sterilization; without high-volume terminal sterilization the risk to patients has the potential to increase significantly. Hence, to provide sterile medical products for patients, the sterility assurance community needs to develop innovative strategies to work through these challenges.

The second challenge is the growing number of sensitive combination products and biopharmaceutical products that are not easily sterilized with traditional terminal sterilization approaches. Some of these products are challenging to aseptically process with traditional approaches due to the size of the molecules as compared to the pore size of the filters typically used during the aseptic process. If filtration is not used during the aseptic process, the manufacturing handling procedures are extremely critical to delivering a product with the appropriate level of microbiological quality. This drives the need for sterilization innovation to ensure that innovative new combination products make it to the market. This emphasizes the need for sterility assurance professionals to be well-grounded in sterility assurance principles to find innovative new sterility assurance solutions.

Terminal sterilization requirements for sterile product have been the driving force for a key recent innovation in the standards that could benefit both terminally sterilized and aseptically processed product. EN 556-1 provides a very clear framework for designating a terminally sterilized medical device as “STERILE.” Highlighted portions of EN 556-1 section ‘Education’ provide the framework,

“Section ‘Education’: For a terminally-sterilized medical device to be designated ‘STERILE,' **the theoretical probability of there being a viable micro-organism present on/in the device shall be equal to or** **<1** **×** **10**^**−**6****^.
Note: Permission for acceptance of a probability greater than that specified in section ‘Education’ may be sought through the appropriate regulatory bodies. Such permission depends on the individual situation, including consideration of the risk management activities (see, for example, EN ISO 14971) undertaken by the manufacturer of the medical device.”

In practice, the EN 556-1 requirement for designating a terminally sterilized product as sterile has been summarized as, “**the sterility assurance level**,^5^ **SAL, shall be** **<10**^**−**6****^”.

Industry practice has been focused on the requirement, not the note following that has the guidance that consideration of use of a probability or a greater SAL than 10^−6^, e.g., 10^−4^, may be acceptable based on consideration of the patient risk. This note is extremely important in EN 556-1 as it provides an alternative way to approach sensitive combination products. A key example of the reason why sensitive combination product cannot be terminally sterilized is due to the requirement to achieve an SAL of 10^−6^. If this SAL cannot be achieved, then industry has defaulted to aseptic processing. However, an alternative might be to utilize an SAL with a probability of >10^−6^, e.g., 10^−4^, that might allow for terminal sterilization without compromising product functionality.

ST67:2003 documented this approach and it has been used since the early 1980's for products that do not come into contact with compromised tissues^6^ and/or could not withstand a sterilization process to achieve a SAL of 10^−6^. While EN 556-1 allows for the potential to utilize greater SALs, e.g., 10^−4^, if justified, it does not provide a means for the justification of a greater SAL, e.g., 10^−4^. This application-oriented shortcoming in EN 556-1 was the rationale for the development of ISO/TS 19930:2017. The significant achievement with this document was the inclusion of a framework for a risk assessment for products that are not compatible with a terminal sterilization process designed to deliver an SAL of 10^−6^ but that might be compatible with a process designed to deliver an SAL of >10^−6^, e.g., 10^−4^. This new resource for the industry provides important perspective for both the terminal sterilization sector and the aseptic processing sector.

**Figure 1:**
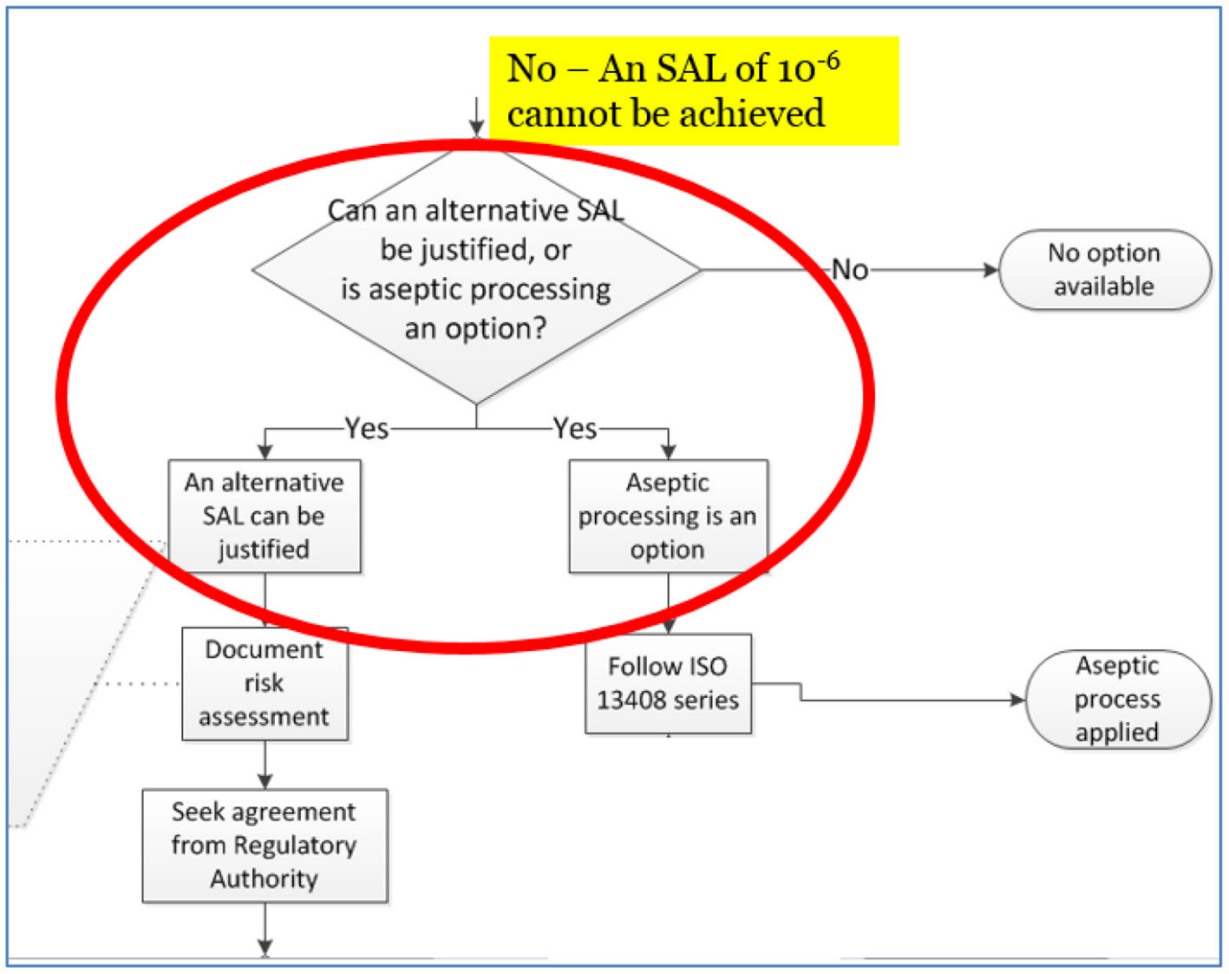
Excerpt from Figure A.1 of ISO/TS 19930:2017, illustration of the sequence of activities in selecting and justifying an SAL >10^−6^, e.g., 10^−4^.

A critical bifurcation in this new guidance document for product that cannot withstand an SAL of 10^−6^ is shown in the Figure 1. The branch on the right, aseptic processing, is well-established and the industry has successfully used this approach for decades (see section ‘Aseptic Processing’). This branch is therefore expected in this decision tree when an SAL of 10^−6^ cannot be achieved. What is not expected is the left branch of the decision tree, on equal footing with aseptic processing while providing a reduced risk is the use of an SAL >10^−6^, e.g., 10^−5^ or 10^−4^. This is typically not thought of as an option to aseptic processing; however, per Figure 1 it should be, since it has been in practice since the 1980's as noted previously.

This gets to the heart of the complexity demanded of sterility assurance professionals in facing the challenges of finding solutions for sensitive combination products. For example, can the risks associated with providing an aseptically produced combination product be compared with those of a terminally sterilized combination product using an SAL >10^−6^, for example, 10^−4^? Does the patient benefit more from one decision branch or the other? ISO/TS 19930 puts both options on equal footing.

This point is important for the aseptic processing community. It provides the appropriate regulatory paradigms for minimizing risk to patient through leveraging terminal sterilization while minimizing deleterious effects on the product. USP <1211> highlights this situation, “An aseptic process followed by a terminal sterilization process provides superior control over the presterilization bioburden, such that the subsequent sterilization process can be designed with less overall lethality, thereby making it possible to substantially extend the use of terminal sterilization to products with greater sensitivity to the applied energy of the process.” A key paradigm for this is risk-based application of bioburden-based terminal sterilization, as discussed in section ‘Aseptic Processing’.

The next, section ‘Terminal Sterilization’, and the tools discussed in section ‘New Paradigms and Tools in Sterility Assurance’, provide insights as well as confidence in using valuable but long-neglected SALs >10^−6^ (e.g., 10^−5^ or 10^−4^).

### Terminal Sterilization

The core sterilization standards that have served the industry well and/or that are being developed are listed in Table 1. As discussed above, terminal sterilization is preferred over aseptic processing. The rationale for this preference can be seen in the foundational sterility assurance principles and current realities that permeate these standards as summarized in section ‘Foundational Terminal Sterilization Principles and Current Realities’. ISO 14937, *Sterilization of health care products—General requirements for characterization of a sterilizing agent and the development, validation, and routine control of a sterilization process*, provides the general structure for extending these principles to non-traditional sterilization modalities, beyond those for which there is a specific standard.

**Table 1:** Standards for validation and routine control of sterilization processes.

**Standard reference**	**Standard title**	**Date of publication**
ISO 14937	Sterilization of health care products **General requirements** for characterization of a sterilizing agent and the development, validation, and routine control of a sterilization process	2009
ISO 11135	Sterilization of health-care products—**Ethylene oxide**—Requirements for the development, validation and routine control of a sterilization process for medical devices	2014 AMD1:2019
ISO 11137-1 ISO 11137-2 ISO 11137-3 ISO/TS 11137-4	Sterilization of health care products Requirements for the development, validation and routine control of a sterilization process for medical devices **Radiation** Part 1: Requirements Part 2: Establishing the sterilization doses Part 3: Guidance on dosimetric aspects of development, validation, and routine control Part 4: Part 4: Guidance on process control	2006/AMD1 2013, AMD2 2019 2013 2017 2020
ISO 17665-1 ISO/TS 17665-2 ISO/TS 17665-3	Sterilization of health care products—**Moist heat** Part 1: Requirements for the development, validation and routine control of a sterilization process for medical devices Part 2: Guidance on the application of ISO 17665-1 Part 3: Product families	2006 2009 2013
EN ISO 20857	Sterilization of health care products—**Dry heat**—Requirements for the development, validation and routine control of an industrial sterilization process for medical devices	2013
EN ISO 25424	Sterilization of medical devices—**Low temperature steam and formaldehyde**—Requirements for development, validation, and routine control of a sterilization process for medical devices	2019
EN ISO 14160	Sterilization of health care products—**Liquid chemical sterilizing agents for single-use medical devices utilizing animal tissues and their derivatives**—Requirements for characterization, development, validation, and routine control of a sterilization process for medical devices	2011

#### Foundational Terminal Sterilization Principles and Current Realities

Why is terminal sterilization preferred over aseptic processing to manufacture sterile product? As noted above, a validated and reproducible (i.e., controlled) methodology of systematically inactivating product bioburden is deemed to provide more confidence than undertakings to eliminate the introduction of microbial contamination during aseptic processing. What is the basis of the confidence in terminal sterilization processes? Three major components of the confidence are the challenge process, the validation approaches, and the ability to reproducibly deliver and demonstrate control of the process.

##### The Challenge Process

Validation of the sterilization process includes demonstration of sterilant delivery to the most difficult location within the processing load and the most difficult location within the product, and demonstration that the process can inactivate microorganisms that are a challenge to the sterilant. The following briefly explore each of these challenges:

*Location within the sterilization processing load*. Validation practice is to identify the location within a routine standard processing load that is the most difficult to deliver the sterilant. This is the location where the minimum parameters are demonstrated to be achieved during routine processing. For radiation sterilization, a single dose across the entire processing load is not achievable, therefore a radiation dose range is defined and validated. The sterilization “minimum dose” achieves the desired SAL and the sterilization “maximum dose” is demonstrated to ensure that the product functions following sterilization. For sterilization modalities such as gas and vapor (e.g., ethylene oxide, chlorine dioxide), a single parameter is not used because multiple parameters (e.g., temperature, humidity, gas concentration, and sterilant exposure time) need to be demonstrated to deliver the desired SAL. For heat modalities (i.e., moist and dry heat), a single parameter is not used because multiple parameters (e.g., temperature and time) need to be demonstrated to deliver the desired SAL. For all sterilization modalities, the standards define that minimum processing parameters must be designed to deliver the desired SAL to the most difficult to achieve location within the processing load, and the maximum parameters are designed to ensure that the product functionality remains acceptable for intended use.

*Location within the product*. Healthcare products are not uniform as they consist of product components made up of different materials, and product design attributes play a role in determining the most difficult to sterilize location. Design attributes that potentially impact the ability to sterilize include an assessment of items like small bore lumens that are hard for a gas sterilant to penetrate, or product components that have high thermal inertia or high density that make it difficult for heat or radiation, respectively, to penetrate. All sterilization modalities standards require that desired lethality, e.g., 10^−6^ SAL, be achieved at the location within the product that provides the greatest challenge to lethality.

*Microbiological challenge to the sterilant*. Sterilization processes are validated either using a biological indicator (BI) or using the product's natural bioburden. If BIs are used, it must be demonstrated that they provide a challenge to the sterilization process greater than the natural bioburden of the product. If product natural bioburden is used, it must be demonstrated that the more highly resistant bioburden organisms are taken into account in the sterilization validation approach, as appropriate. All the sterilization standards require that defined lethality be achieved including microorganisms that provide the greatest challenge to lethality.

The conservativeness of the current realities of the sterilization standards are further amplified based on additional realities of sterilization validation methodologies as discussed in section ‘Validation Approaches’.

##### Validation Approaches

**Figure 2:**
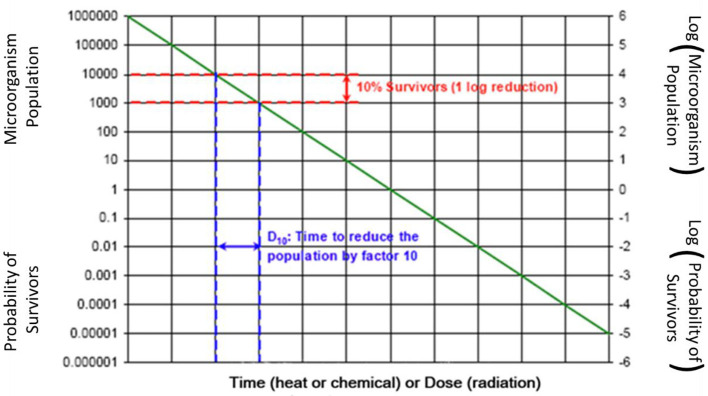
Schematic of traditional terminal sterilization microbial log reduction [(4), Figure 1].

Based upon microbiological characterization of traditional sterilants, a log-linear inactivation rate is assumed with sterilant exposure, per Figure 2. This figure shows the classic curve starting in the upper left corner with one million organisms (e.g., Biological Indicator spores) starting population with no exposure to sterilant. With exposure to sterilant, the microbiological population is reduced logarithmically as shown. Due to ease of use for many traditional medical devices with limited material compatibility concerns, “overkill” validation methodologies have been commonly used. There are also many bioburden-based validation methodologies that provide much more gentle sterilization cycles to minimize material compatibility concerns. The overall conservative nature of terminal sterilization is reviewed along with some useful innovation in this space.

A widely used and explicitly phrased “overkill method half-cycle approach” involves use of one million challenge organism spores in the hardest to kill location in the product and in the load configuration. The duration of exposure is defined to provide lethality or inactivation and then the duration is doubled. This results in at least a 12-log reduction in bioburden. Note: Figure 2 only shows an 11-log reduction in bioburden, from 10^6^ to 10^−5^ colony forming units (cfu). Another “overkill method” allows the calculation of log reduction based on product bioburden results, for example if the product bioburden is consistently <1,000 cfu, only a nine-log reduction in microbial load is required to achieve a 10^−6^ SAL.

Clearly there is significant additional lethality in these purposefully designed overkill sterilization validation methods. There can also be additional conservative lethality provided in bioburden-based sterilization validation methods. This can be seen in a radiation sterilization validation example, a category of validations often regarded as having minimal conservativeness. The ISO 11137-2 standard for radiation sterilization provides validation methods that are bioburden-based. A product's natural bioburden is challenged against a standard microbial distribution from the industry that is the basis of the method, the “standard distribution of resistances,” SDR, which inherently includes subpopulations of highly resistant organisms. This results in an even more conservative approach in many cases. Sub-lethal radiation dose(s), based on the level of product bioburden, is applied to the product to determine if the natural product bioburden has a resistance to radiation lethality equal to or less than the SDR. All this to say, a specific radiation dose is defined that will provide to the product the desired assurance of sterility, e.g., 10^−6^ SAL.

**Figure 3:**
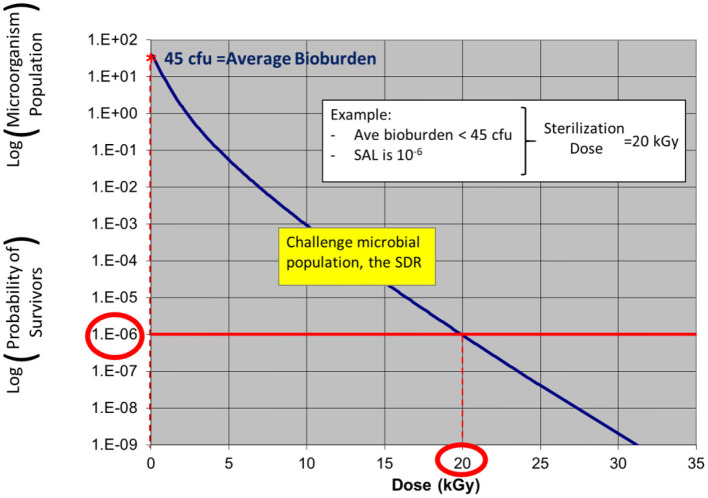
ISO 11137 process definition—establishing a radiation sterilization dose of 20 kGy corresponding to product bioburden <45 cfu. (note: due to the nature of the SDR, the traditional straight log-linear curve is not applied as log-linear is only applicable for a single microorganism challenge).

**Figure 4:**
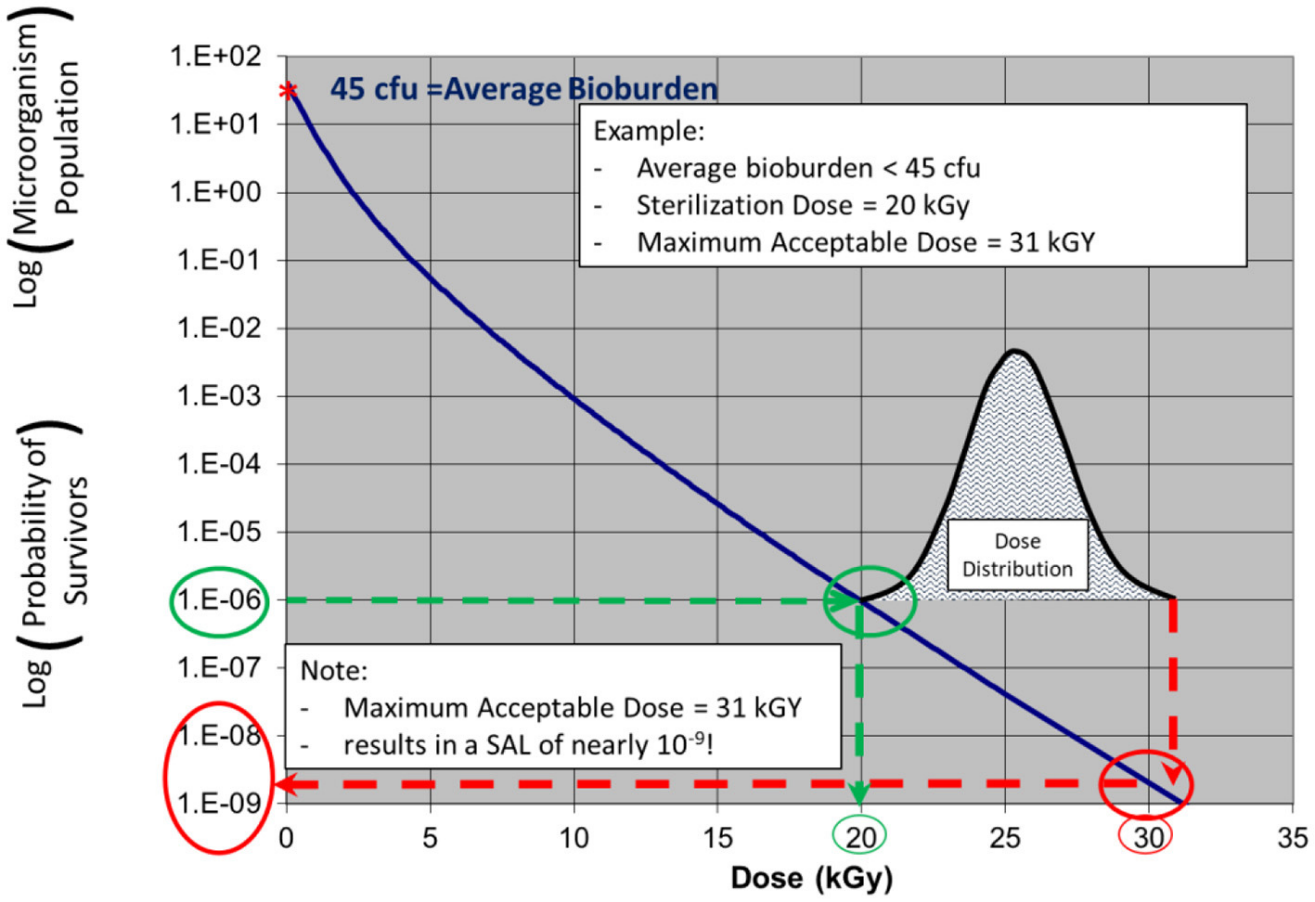
ISO 11137 performance qualification—a relatively narrow dose mapping distribution highlighting significant additional lethality (conservativeness) to much of the product.

Note: in ISO 11137-2 the SDR is presented in table form. The starting population in this example shows a product bioburden of 45 cfu. As dose is increased up to 20 kGy, the bioburden level is reduced to a level of one organism in one million, an SAL of 10^−6^.

In the examples in Figures 3, 4, a product with bioburden <45 cfu is validated with a sterilization dose of 20 kGy. The SDR is shown graphically in Figure 3.

The additional lethality, or inherent conservativeness, is seen in Figure 4 which shows the results of a narrow (conservative) dose mapping distribution, the output of ISO 11137 Performance Qualification. The dose received by the product ranges from 20 to 31 kGy. At 31 kGy, the sterility assurance level provided to the part of the product with this dose is nearly one in a billion, 10^−9^ SAL. Hence, for the dose range of 20–31 kGy, the product will possess SALs ranging from 10^−6^ to 10^−9^. Clearly this bioburden-based validation method is conservative.

This same conservativeness in the delivery of the sterilization process is also observed when using all of the current terminal sterilization processes due to the distribution of critical process parameters—depending on the sterilization modality—such as sterilant, temperature, humidity, time, etc. The combination of a rigorous process challenge combined with the explicit or inherent additional lethality of the processes are key parts of the reason that terminal sterilization is preferred over aseptic processing.

Another approach to innovatively validating terminal sterilization solutions for sensitive product relates to minimizing sample size and is illustrated with Method VD_Max_ for radiation sterilization validations per ISO 11137. It is an example of improving standards to solve issues of terminal sterilization of infrequent batches of sensitive biological items. Method VD_Max_ was intended for substantiating a preselected dose along with providing the potential of lowering the material exposure to sterilization agent if, for example, Method VD_Max_^15KG^ is selected. Regardless of the SAL, the development of this method has provided solutions to small product batches e.g., biological tissue allografts. Because other methods (Methods 1, 2A, and 2B) of this ISO document are not applicable due to the huge sample number required from a single batch for bioburden and dose setting, the use of Method VD_Max_ for small batches was of great value for tissue allograft producers and can be applicable to combination products.

##### Reproducibility

The final reason, and potentially most important, why terminal sterilization is a preferred method over aseptic processing is that the process is validated to reproducibly deliver and demonstrate control of the process. One of the final steps in the sterilization standards (Table 1) is defining the routine process monitoring and control measures required for sterilization. Parameters of the process that were validated to provide lethality are linked with process indicators that can be monitored—such as dosimeters or BIs or process parameters themselves. This allows for assured control and confidence in the sterilization process with its associated robustness. This robustness has been documented in decadal studies by the Center for Disease Control and Prevention (CDC) showing no hospital infections linked to terminally sterilized product (5–7). In addition, routine monitoring of manufacturing controls to ensure that the product bioburden number and resistance do not increase over time provides support that the process remains in the validated state and is a critical component to support this reproducibility. Decadal studies using routine maintenance of the sterilization validation in combination with routine manufacturing controls has demonstrated that terminal sterilization of single use medical products has been maintained successfully worldwide.

### Aseptic Processing

Aseptic processing is both an art and a science. As noted previously, the aseptic processing definition^7^ is “handling of sterile product, containers, and/or devices in a controlled environment in which the air supply, materials, equipment, and personnel are regulated to maintain sterility.” This means excluding microorganisms to maintain sterility. Exceptional control is required, especially if manual interventions are required. This is addressed in the ISO and other standards in Table 2. This is followed by a brief discussion of best practices.

**Table 2:** Selected standards and guidance documents for aseptic processing.

**Standard reference**	**Selected standard title and guidance documents**	**Date of publication**
EN ISO 13408-1	Aseptic processing of health care products—Part 1: General requirements	2011/AMD1 2013
EN ISO 13408-2	Aseptic processing of health care products—Part 2: Filtration	2018
EN ISO 13408-3	Aseptic processing of health care products—Part 3: Lyophilization	2011
EN ISO 13408-4	Aseptic processing of health care products—Part 4: Clean-in-place technologies	2011
EN ISO 13408-5	Aseptic processing of health care products—Part 5: Sterilization-in-place	2011
EN/ISO 13408-6	Aseptic processing of health care products—Part 6: Isolator systems	2011
EN ISO 13408-7	Aseptic processing of health care products—Part 7: Aseptic qualification of solid medical devices and combination medical devices	2015
ISO 18362	Manufacture of cell-based health care products—Control of microbial risks during processing	2016
ISO 17665-1	Sterilization of health care products—Moist heat—Part 1: Requirements for the development, validation and routine control of a sterilization process for medical devices	2006
USP <1211>	Sterility assurance	2019
USP <1229>	Sterilization of compendial articles	2013
USP <1229.1>	Steam sterilization by direct contact	2013
USP <1229.2>	Moist heat sterilization of aqueous liquids	2019
USP <1229.4>	Sterilizing filtration of Liquids	
USP <1229.6>	Liquid phase sterilization	2018
USP <1229.7>	Gaseous sterilization	2018
USP <1229.8>	Dry heat sterilization	2018
USP <1229.10>	Radiation sterilization	2014
USP <1229.11>	Vapor phase sterilization	2015
USP <1229.12>	New sterilization methods	2016
USP <1229.13>	Sterilization in-place	2016
USP <1229.14>	Sterilization cycle development	2017
EMA/CHMP/CVMP/ QWP/850374/2015	Guideline on sterilization of the medicinal product, active substance, excipient and primary container	2019
ISO/TS 19930	Guidance on aspects of a risk-based approach to assuring sterility of terminally sterilized, single-use health care product that is unable to withstand processing to achieve maximally a sterility assurance level of 10^−6^	2017
PDA No. 1	Technical Report No. 1, Revised 2007 (TR 1) Validation of moist heat sterilization processes cycle design, development, qualification and ongoing control	2007
PDA No. 3	Technical Report No. 3 (Revised 2013) Validation of dry heat processes used for depyrogenation and sterilization	2013
PDA No. 13	Fundamentals of an environmental program	2014
PDA No. 22	Process simulation for aseptically filled products	2011
PDA No. 44	Quality risk management for aseptic processes	2008
PDA No. 54-5	Quality risk management for the design, qualification, and operation of.	2017
PDA No. 60	Process validation: a lifecycle approach	2013
PDA No. 61	Technical Report No. 61 Steam in place	2013
PDA Parts 1 and Part 2	Aseptic processing points to consider	2015/2016
FDA 2004 Aseptic Guidance	Guidance for industry. Sterile drug products produced by aseptic processing-current good manufacturing practice	2004
EU Annex 1	Manufacture of sterile medicinal products	2008

#### Core Standards Related to Aseptic Processing

The medical device sector often looks to ISO standards. The aseptic processing community often looks to pharmacopeial standards. In cases like radiation sterilization, there is good alignment. There is less alignment with other overlapping sterilization approaches, e.g., ethylene oxide.

ISO standards for aseptic processing define risk-based requirements for the manufacture of aseptically processed sterile product. It starts with characterization and control of the manufacturing environment and equipment; where cleanroom classification (see section ‘Cleanrooms and Microbiological Control’) is a key component. Personnel training, gowning, and general health requirements are also addressed. Requirements for manufacturing of product using aseptic technique are provided. Process simulation requirements are provided to qualify the process, with tests of sterility as demonstration of acceptable simulations.

Aseptic processing often utilizes components that are terminally sterilized. ISO and United States Pharmacopeia (USP) provide requirements for terminal sterilization. As noted above, they are aligned well in some cases.

The European Medicines Agency (EMA) recently updated a decision tree for sterilization of medicinal product.^8^ The Parenteral Drug Association (PDA) also provides widely utilized guidance for aseptic processing.

#### Current Aseptic Processing Realities

Aseptic processing best practices have been published (1, 2). They include a prudent strategy focused on aseptic processing and sterility by design, using a powerful line-of-sight process control approach and leading to robust user requirement specifications (URS), and development. Practical and best aseptic practices and environmental monitoring cleanroom design approaches include aseptic barrier systems, e.g., isolators, and critical utilities. Practical process simulation design is also best practice. Quality risk management and risk based critical thinking are required as well as consideration of new technology and thinking in the field of aseptic processing.

Best practices and best technology can minimize personnel interventions and optimize quality to levels that exceed those in the aseptic processing standards (ISO 13048) that quantify certain aspects of the standard. A potential best practice in the aseptic processing sector is the use of terminal sterilization following aseptic processing. Although this is sometimes referred to as adjunct processing; it is called “post aseptic processing terminal sterilization” in USP <1211>. USP <1211> highlights that it is synergistic for providing sterility while minimizing effects on the product. The main reason to use aseptic processing is to minimize deleterious effects of the sterilant on the product. Another option might be post aseptic fill lethal treatment, which could employ heat treatment processes (e.g., 80°C) with heat histories much lower than moist heat terminal sterilization processes to significantly reduce risks with aseptic processing without degrading products (8). Terminal sterilization provides reproducible active reduction of microbial contamination on product, but can have negative material effects. If aseptic processing is used in conjunction with terminal sterilization, it can be possible to keep pre-sterilized bioburden sufficiently low enough to enable gentler sterilization conditions, which can have a minimal negative functional effect on product.

Industrial subject matter experts (SMEs) can take advantage of this intersection between terminal sterilization and aseptic processing in developing novel products. In ISO/TS 19930, Clause 7 (Table 3), strategies are provided to achieve an SAL of 10^−6^ prior to either using alternative SALs or aseptic processing. Item “(i)” in this list is, “Applying a microbiological inactivation process as an adjunct to aseptic processing.” Along with the generic example provided in ISO/TS 19930 (see Example 8 in Table 3), Agalloco has written a series of three papers fleshing out the logic, science and implementation for moist heat sterilization (9–11).

**Table 3:** ISO/TS 19930, clause 7, strategies to achieve a maximal SAL of 10^−6^.

(a) Changing materials to enable product, including the sterile barrier system, to withstand the processing conditions necessary to achieve maximally a SAL of 10^−6^. (b) Redesigning product to enable it to withstand the processing conditions necessary to achieve maximally a SAL of 10^−6^. (c) Changing the presentation of product to the sterilization process to reduce the extent of treatment. (d) Changing conditions in the sterilization process to reduce the detrimental effects on product. (e) Considering an alternative method of establishing the sterilization process if using a conservative overkill approach to process definition does not achieve maximally a SAL of 10^−6^ without a detrimental effect on product. A different approach to establishing the sterilization process might provide a process with less severe conditions that can achieve a maximal SAL of 10^−6^. EXAMPLE 1 Use of a combined bioburden-biological indicator approach to process definition rather than an overkill approach for EO sterilization (see ISO 11135) EXAMPLE 2 Use of a bioburden-based approach for dry or moist heat sterilization (see ISO 17665-1 or ISO 20857) instead of use of a standard time-temperature combination. EXAMPLE 13 Use of Method 2 instead of Method VDmaxSD to establish the sterilization dose for radiation sterilization (see ISO 11137-2). (f) Considering another sterilization process that could achieve maximally a SAL of 10^−6^. EXAMPLE 4 If the temperature or humidity required for EO sterilization is an issue, then a radiation sterilization process could be considered. EXAMPLE 5 If radiation effects in the product are an issue, then an EO, dry heat or moist heat sterilization process could be considered. EXAMPLE 6 If radiation, EO, dry heat or moist heat sterilization process are not appropriate, sterilization processes for which currently there are no specific standards for validation and routine control, such as exposure to hydrogen peroxide vapor or chlorine dioxide gas, could be considered. (g) Reducing and controlling product bioburden to allow the use of sterilizing conditions that have less detrimental effect. EXAMPLE 7 For radiation sterilization, reducing the bioburden might allow use of a lower sterilization dose. (h) Applying microbiological inactivation processes sequentially. (i) Applying a microbiological inactivation process as an adjunct to aseptic processing. It might be possible to deliver a terminal sterilization process by applying a microbicidal process that on its own would not achieve a maximal SAL of 10^−6^ following an aseptic process. EXAMPLE 8 This scenario is illustrated by aseptic filling a fluid and subsequently applying a moist heat process. The adequacy of the aseptic process is demonstrated by process simulation with a contamination rate of 1 non-sterile unit in 10,000 units filled. A moist heat process of 115^°^C for 15 min is selected based as the greatest time-temperature combination that the product can withstand, on the assumption that any contaminating microorganisms following the aseptic process are not as resistant to moist heat as the standard reference microorganisms for moist heat sterilization. This could be confirmed by culturing the microorganisms from the positive process simulation and determining their moist heat resistance.

### Packaging Sterile Barrier Systems

ISO standards for packaging sterile barrier systems are in Table 4.

**Table 4:** Standards for packaging sterile barrier systems.

**Standard reference**	**Standard title**	**Date of publication**
ISO 11607-1	Packaging for terminally sterilized medical devices. Requirements for materials, sterile barrier systems and packaging systems	2020
ISO 11607-2	Packaging for terminally sterilized medical devices. Validation requirements for forming, sealing and assembly processes	2020

ISO 11607 packaging standards for terminally sterilized medical devices provide general requirements for quality systems, risk management, sampling, test methods, and documentation. Microbial barrier, sterilization compatibility, labeling, and storage/transport requirements for materials and package components are provided. Design and development requirements are delineated, along with those for aseptic presentation. Packaging system performance and stability requirements are then discussed along with validation [installation qualification (IQ), operational qualification (OQ), and performance qualification (PQ)] requirements.

Current practices for the performance of packaging validations are not able to demonstrate that the sterile barrier system maintains the desired SAL, e.g., 10^−6^, over the shelf-life of the product. In order to demonstrate the maintenance of the desired SAL, approximately three million samples would be required to demonstrate with attribute data (e.g., a pass/fail leak test) at the end of shelf-life with reasonable confidence. Sample sizes are discussed in section ‘Packaging Sterile Barrier Systems’ in discussing quantifiable aspects of the standard.

Maintenance of sterility is required on container closures for aseptically processed product and sterile barrier packaging for terminally sterilized medical devices. Sterility assurance and/or packaging professionals need to optimize technologies; process validation, including test methods and design validation; samples sizes with statistical considerations; and new developments across the industry sector. Sample size is a key part of the analysis in section ‘Aseptic Processing’ to quantify certain aspects of the ISO 11607 standard.

### Cleanrooms and Microbiological Control

Cleanrooms and microbiological control are foundational to all manufacturing processes of healthcare products. ISO standards are given in Tables 5, 6. The extent of the controls is adjusted based upon the type of manufacturing processes selected. Cleanrooms, microbiological control, and testing cannot be ignored as they are foundational mechanisms to provide confidence in quality product and to achieve sterility assurance requirements. Analogous procedures required for hospitals to avoid hospital acquired infections is discussed in section Healthcare Setting and Device Reprocessing and highlights the critical need for and complexity in achieving excellence in this area.

**Table 5:** Standards for cleanroom manufacturing space control and monitoring^9^.

**Document**	**Title**	**Date**
ISO 14644-1	Classification of air cleanliness by particle concentration	2015
ISO 14644-2	Monitoring to provide evidence of cleanroom performance related to air cleanliness by particle concentration	2015
ISO/ANS 14644-3	Test methods	2005
ISO 14644-3	Test methods	2019
ISO 14644-4	Design, construction, and start-up	2001
ISO 14644-5	Operations	2004
ISO 14644-7	Separative devices (clean air hoods, gloveboxes, isolators, minienvironments)	2004
ISO 14644-8	Classification of air cleanliness by chemical concentration (ACC)	2013
ISO 14644-9	Classification of surface particle cleanliness	2012
ISO 14644-10	Classification of surface cleanliness by chemical concentrations	2013
ISO 14644-12	Specifications for monitoring air cleanliness by nanoscale particle concentration	2018
ISO 14644-13	Cleaning of surfaces to achieve defined levels of cleanliness in terms of particle and chemical classifications	2017
ISO 14644-14	Assessment of suitability for use of equipment by airborne particle concentration	2016
ISO 14644-15	Assessment of suitability for use of equipment and materials by airborne chemical concentration	2017
ISO 14644-16	Code of practice for improving energy efficiency in cleanrooms and clean air devices	2019
ISO/FDIS 14644-17	Particle deposition rate applications	FDIS2020

**Table 6:** Standards for microbiological methods to support microbiological control.

**Standard reference**	**Standard title**	**Date of publication**
EN ISO 11737-1	Sterilization of medical devices. Microbiological methods. Determination of the population of microorganisms on products	2018
EN ISO 11737-2	Sterilization of medical devices. Microbiological methods. Tests of sterility performed in the validation of a sterilization process	2019

### Healthcare Setting and Device Reprocessing

When considering the broader picture of patient risk related to sterility assurance, a critical topic is the healthcare setting itself, including healthcare associated infections (HAI), along with device reprocessing. Many of the cleanroom concepts and aseptic processing concepts discussed above apply in the operating theater and throughout a hospital or clinic. Terminal sterilization of devices that can be reused/reprocessed often occurs in hospital central sterilization services areas, with reprocessing of surgical tools and some medical devices being common and potentially high-profile due to associated patient risks. A recent review (7) highlights best practices and the current reality that HAI is an important issue for patients in healthcare facilities.

HAI^10^ rates range from 3.5 to 19.1% depending on geography. These are staggering rates that impact patient well-being costs. Risk related to HAI includes the basics of good personnel practices; difficult care associated with long-term invasive devices such as blood ports and urinary tract catheters; and antibiotic resistance organisms (7). The sterility assurance professional must be able to put this critical issue into perspective in developing innovative sterility assurance solutions for innovative products that will serve patients well in coming decades.

## New Paradigms and Tools in Sterility Assurance

The context provided above in section Sterility Assurance Standards and Current Realities are principles and summaries of current realities in a number of industry sectors that shape the landscape for building solutions. Sterility assurance professionals within individual sectors have an understanding of the principles, standards and paradigms related to their sector, but, in most cases, not as deep an understanding of other practices used by other sectors. Cross-sector discussions and potential solutions have therefore been difficult to objectively facilitate. Another reason, however, for the difficulty in discussing concepts cross-sector is the lack of a common vocabulary for these discussions particularly with sterility terms such as sterility assurance level (SAL) and probability of a non-sterile unit (PNSU). To break down these barriers, a non-traditional approach was published (12) with a potential new term PNSU# that focuses solely on the quantifiable aspects of sample sizes defined in standards used in a given industry sector. The goal of this new term is to help the industry stop being enamored with sample sizes and focus on the entirety of sterilization assurance and microbial quality of the product. The goal of section ‘Spectrum of Microbiological Quality for Product’ is to help sterility assurance professionals become familiar with this new approach and terms.

Before we dive into the new approach and terms, it is important to discuss ISO/TS 19930 in the next section that can foundationally shape the discussion around cross-sector opportunities. Aseptic processing, as shown previously in Figure 1, is one of two options for products that have materials that are not compatible with sterilization solutions at an SAL of 10^−6^. The second option, greater SALs than 10^−6^, has not been recognized by the sterility assurance community across multiple industry sectors around the world. Digesting the implications of this guidance can help shape the conversation around cross-sector assurance of sterility solutions.

Part of the reason for the disconnect around the world has to do with the designation of medical devices. In the United States, some products used in healthcare practices that do not contact compromised tissue have been designated as medical devices (for example gowns and drapes). They have a history for many decades of being sterilized at greater SALs than 10^−6^ (e.g., 10^−3^) and still being labeled as “STERILE.” However, these same products sold in many other countries around the world are not categorized as medical devices and in some cases are not deemed to require a “STERILE” label claim (13).

Understanding the source of the cross-sector differences and collaborating to have a common understanding across the different sectors will help to break down the barriers.

### When an SAL of 10^–6^ Is Not Possible—ISO/TS 19930

As discussed previously and illustrated in Figure 1, the industry has developed guidance to apply SALs >10^−6^ (e.g., 10^−4^ as permitted in the note to the requirements in section ‘Education of EN 556-1’) as an equivalent option to aseptic processing. ISO/TS 19930 frames the preferred approach to providing sterile product, terminal sterilization, but with greater SALs, e.g., 10^−5^ or 10^−4^, as an equally viable approach to be assessed as an equally viable approach as aseptic processing (14).

In light of the guidance provided in ISO/TS 19930 that puts the aseptic processing sector in the same bifurcation picture as the terminal sterilization sector using SALs >10^−6^, e.g., 10^−4^, it seems imperative that cross-sector collaboration progress rapidly. One of the barriers to such collaboration is misunderstanding due to lack of common terms about quantification of sterility assurance risk. PNSU# is a potential new term to facilitate this dialogue, and to help to put sterility assurance options into perspective, in particular the quantifiable aspects of each sector (section Spectrum of Microbiological Quality for Product). Alternatively, another way to think of this would be to understand the level of microbiological quality desired, and to then understand where the terminal and aseptic processes overlap (section ‘Microbiological Quality/Microbiological Risk’).

### Spectrum of Microbiological Quality for Product

**Figure 5:**
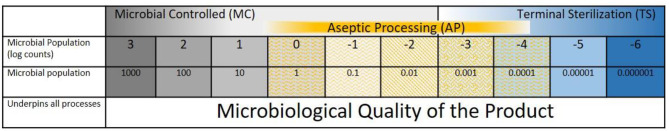
Spectrum of microbiological quality of product.

Microbiological quality is a phrase that has recently come into use because of the challenges previously discussed (i.e., the growing number of products that may not be capable of being terminally sterilized or aseptically filtered). This phrase is intended to help understand both the span of controls possible and the extent of the microbiological controls (e.g., chemistry, aseptic processing or terminal sterilization) necessary to deliver the final finished product to meet patient needs. The microbiological quality (Figure 5) can span an understanding of the product from the beginning of the manufacturing process (e.g., raw materials bioburden) to the completion of the manufacturing process to achieve the desired end point or label claims (e.g., “STERILE”).

The microbiological quality of the raw materials or components, upon receipt at the manufacturer, might be tested to determine the microorganism number (i.e., bioload or bioburden) and types (i.e., defined by gram stain, or genus and species identification) that are present. An understanding of the bioburden of the starting production raw materials provides the direction for the sterility assurance professionals to determine the controls to be taken throughout the production process. The new paradigm shift is to understand to what extent do controls need to be put in place to assure that the final finished product achieves the desired end point. The desired end point might be the reduction of the number and types of microorganisms present on the final product. The reduction of the number and types of microorganisms typically includes an understanding of the types of microorganisms present in the raw materials and the use of agents to inhibit the proliferation of organisms: these are referred to as microbiologically/microbial controlled products. If products are not capable of being aseptically produced through filtration (e.g., due to pore size constraints) or terminal sterilization (e.g., due to detrimental impact to sensitive raw materials), then the product may need to be delivered to the patient in a microbiologically controlled state. The level of microbial control would depend on the patient risk and/or the ability to assure the reproducibility of the microbiological quality of the product. As depicted in Figure 5, this may span the level of 10^3^ microorganisms to 10^−2^ probability of a product having microorganisms.

The other end of the microbiological quality span on the spectrum is the use of Terminal Sterilization to inactivate microorganisms. For products that receive a sterilization process at the end of the manufacturing process, these products are designed and validated to deliver an SAL of 10^−3^, 10^−4^, 10^−5^, or 10^−6^. By design, the aseptic processing of products falls between the microbiologically controlled products and the terminally sterilized products from a microbiological quality standpoint. For products that are aseptically produced, these products are designed and validated typically to deliver a probability of non-sterility of 10^−3^ (or 1 in 1,000).

This spectrum of microbiological quality of product shows how the different levels of control can overlap between the categories of “Microbial Controlled” and “Aseptic Processing,” and between “Aseptic Processing” and “Terminal Sterilization.”

### Quantifiable Portions of Sterility Assurance Standards—PNSU#

The quantification of sterility assurance in the terminal sterilization sector of the healthcare industry is well-defined through the use of the term sterility assurance level, SAL. The term is defined by ISO in their vocabulary document^11^:

**sterility assurance level, SAL—ISO 11139**
probability of a single viable microorganism occurring on an item after sterilization.
Note 1 to entry: It is expressed as the negative exponent to the base 10.

In considering cross-sector solutions to sterility assurance challenges, it can be noted that this definition explicitly restricts usage of the term for aseptic processing since the probability is defined to be after sterilization. It is also not easily applied to the packaging sector in assuring sterility over the shelf-life of a product.

The aseptic processing sector of the healthcare industry sterilizes many components feeding into aseptic processes. The term SAL is appropriately applied to these components. In addition, the aseptic processing sector has used the more intuitive term probability of a non-sterile unit, PNSU, synonymously with SAL. Interestingly, the term PNSU is not in the ISO 11139 vocabulary document. The Parenteral Drug Association, PDA,^12^ defined PNSU in a way that also only applies after a sterilization process,

**probability of a non-sterile unit (PNSU)—PDA TR 1 definition**
The number that expresses the probability of occurrence of a non-sterile unit after exposure to a sterilization process. Within the pharmaceutical industry, a design end point better than or equal to the probability of one non-sterile unit in a million units is expected, i.e., PNSU ≤ 10^−6^ [Synonym: Sterility Assurance Level (SAL)].

An intuitive definition was recently used in an ISO guidance document^13^ and does not restrict usage of the term to product after a sterilization process,

**Probability of a non-sterile unit (PNSU)—intuitive definition**
probability of one or more microorganisms being present on a product item in a population of items.

This definition has the potential to be used in the terminal sterilization, aseptic processing and packaging sectors of the healthcare industry which would be very useful in discussing cross-sector sterility assurance challenges. However, there remains a problem toward this end. The problem centers around the expectation, explicitly stated in the PDA TR 1 definition of PNSU above, of a PNSU of <10^−6^. As discussed in section ‘Sterility assurance principles and Current Realities’ and ‘when an SAL of 10^−6^ Is Not Possible—ISO/TS 19930’, this expectation is not always required for patient safety, and terminal sterilization standards explicitly define pathways for using SAL values >10^−6^, e.g., 10^−4^, in those risk-based situations agreed by national regulatory bodies.

The problem of the 10^−6^ expectation for PNSU is highlighted in the packaging sterile barrier system sector of the healthcare industry. In order to quantitatively and statistically demonstrate that a packaging system maintains a PNSU of 10^−6^ with 95% confidence over the shelf-life of a product, one would need to test approximately three million packages (2,995,731). This is clearly not reasonable, which leads to the problem in quantifying the sample sizes used in the packaging sector. A very responsible approach in a package sterile barrier system validation, which is not feasible with all products, is to test 3,000 units for package integrity. If zero failures occur, the point estimate of sterility assurance is zero. This might be perceived as providing confidence in the packaging system. However, the 95% confidence bound of testing 3,000 units with zero failures is 10^−3^, clearly significantly greater than, and falling significantly short of, an expectation of 10^−6^.

Does this lead to the conclusion that a packaging system validated with 3,000 package integrity tests does not maintain an SAL or PNSU of 10^−6^? Absolutely not. Packaging validations with sample sizes of 3,000 units and less have served patients well for decades. The uncomfortable reality, however, is that this sample size is also not an endorsement that the package system is adequate, perhaps meeting the untestable expectation that it maintains an SAL or PNSU of 10^−6^. The endorsement of adequacy of the packaging system comes only through compliance to a rigorous approach to package sterile barrier system development and validation such as provided in ISO 11607 (see Table 4). When this type of testing is combined with the entirety of the ISO 11607 standard, there is high confidence in the maintenance of sterility through the shelf-life of the product. Note: ISO 11607 provides for confidence in maintenance of sterility through a robust sequential development and validation process (15).

There is an analogous situation with process simulation samples sizes in aseptic processing validations. Process simulation acceptance requirements for four of the six scenarios listed in Table 7 of ISO 13408-1 (see Table 5 above) are zero out of 30,000, 15,000, 3,000, and 100. All four of these results have sterility assurance point estimates of zero. As in zero failures in a package integrity test, this might be perceived as providing confidence in the aseptic system. However, the 95% confidence bound of testing 30,000–100 samples with zero failures ranges from 10^−4^ to 10^−2^, clearly significantly greater than, falling significantly short of, an expectation of 10^−6^ as in the case of package test results. And per the packaging sample size discussion above, to achieve an assurance of sterility value of 10^−6^ with 95% confidence, some 3,000,000 process simulation samples would be required.

**Table 7:** Standards related to designating medical devices as sterile.

**Standard reference**	**Standard title**	**Date of publication**
EN 556-1	Sterilization of medical devices—Requirements for medical devices to be designated “sterile”—Part 1: Requirements for terminally sterilized medical devices	2001/AC: 2006
EN 556-2	Sterilization of medical devices. Requirements for medical devices to be designated “STERILE.” Requirements for aseptically processed medical devices	2015
ANSI/AAMI ST 67	Sterilization of health care products—Requirements and guidance for selecting a sterility assurance level (SAL) for products labeled “sterile”	2019
ISO TS 19930	Guidance on aspects of a risk-based approach to assuring sterility of terminally sterilized, single-use health care product that is unable to withstand processing to achieve maximally a sterility assurance level of 10^−6^	2017
ANSI/AAMI ST67	Sterilization of health care products—Requirements and guidance for selecting a sterility assurance level (SAL) for products labeled “sterile”	2017

Note: two of the six process simulations requirements in Table 7 of ISO 13408-1 permit one failure, out of more than 30,000 and 15,000 samples. These requirements lead assurance of sterility point estimates between 10^−5^ and 10^−4^ and 95% confidence intervals between 10^−4^ and 10^−3^.

Similarly to package validation testing, one can ask if this leads to the conclusion that an aseptic process validated with a process simulation sample size of 30,000 does not provide an adequate assurance of sterility since it does not achieve the expectation of a PNSU of 10^−6^? Absolutely not. Aseptic processing validations with process simulation sample sizes of 30,000 units and less have served patients well for decades. The uncomfortable reality, however, is that these sample sizes are also not an endorsement that the aseptic process validation is adequate, perhaps meeting the untestable expectation a PNSU of 10^−6^. The endorsement of adequacy of the aseptic process comes only through compliance to a rigorous approach to validation of an aseptic process such as provided in ISO 13408. It requires compliance to the entirety of the standard. These sample sizes, when combined with the entirety of ISO 13408 or other aseptic processing standards, provide high confidence in the manufacture of sterile product. Note: ISO 13408-1 and other industry standards provide for confidence in sterile aseptically processed product through robust characterization and control of the manufacturing environment and equipment; personnel training, gowning and general health requirements; and manufacturing aseptic technique. Process simulation requirements are provided as a part of the process to qualify the process, with appropriate tests for sterility (2).

#### Definition of PNSU#

PNSU# was a proposed term to complement, not replace, PNSU and SAL. This term would provide a common language for all sectors. It is intended to provide a language for discussion of the statistical realities of sample sizes used in all healthcare sectors divorced from the discussion of assurance of sterility provided through compliance to standards available in each sector. A rigorous definition of PNSU# was not provided in the publication where it was introduced (12). It was introduced as a tool to focus on one dimension of the sterility assurance toolkit, the quantifiable aspects of the sample sizes used in different sterility assurance sectors and standards. It was repeatedly emphasized that sterility assurance comes from application of all dimensions of a given standard in a given sector. It was also repeatedly emphasized that the specific sample sizes of a given standard do not provide assurance of sterility. Hence the calculation of PNSU# related to sample sizes only tells just one dimension of the story but does not speak to assurance of sterility. Again, it is a supplement to the terms SAL and PNSU, not a replacement term.

**PNSU#—initial definition for industry discussion**
**the point estimate and confidence interval calculated by the sample sizes used in a sterility assurance related validation exercise**

Note: PNSU# is not a measure of sterility assurance; sterility or maintenance of sterility is achieved through compliance to the entire body of work in a sterility assurance standard [see (12)].

#### Application of PNSU#

For the purposes of clarity in understanding the application of this new term, PNSU# will be first discussed relative to the quantifiable aspects of packaging sterile barrier system validations and aseptic processing. The use of the term is simple and clear in these cases. This is followed by application of PNSU# to terminal sterilization which has significantly greater complexity.

Packaging sterile barrier system validations and aseptic processing are the simpler cases because the sample sizes used in this analysis generate non-parametric data (attribute data), e.g., simple pass-fail results, as opposed to parametric data (variable data) involving numeric values as results. An accessible introduction to the relevant statistical concepts and a detailed description of the methodology for application in each industry sector is provided in Speck et al. (12). The discussion below only focuses on the outputs of the analysis from Speck et al. (12).

#### Packaging Sterile Barrier Systems

**Figure 6:**
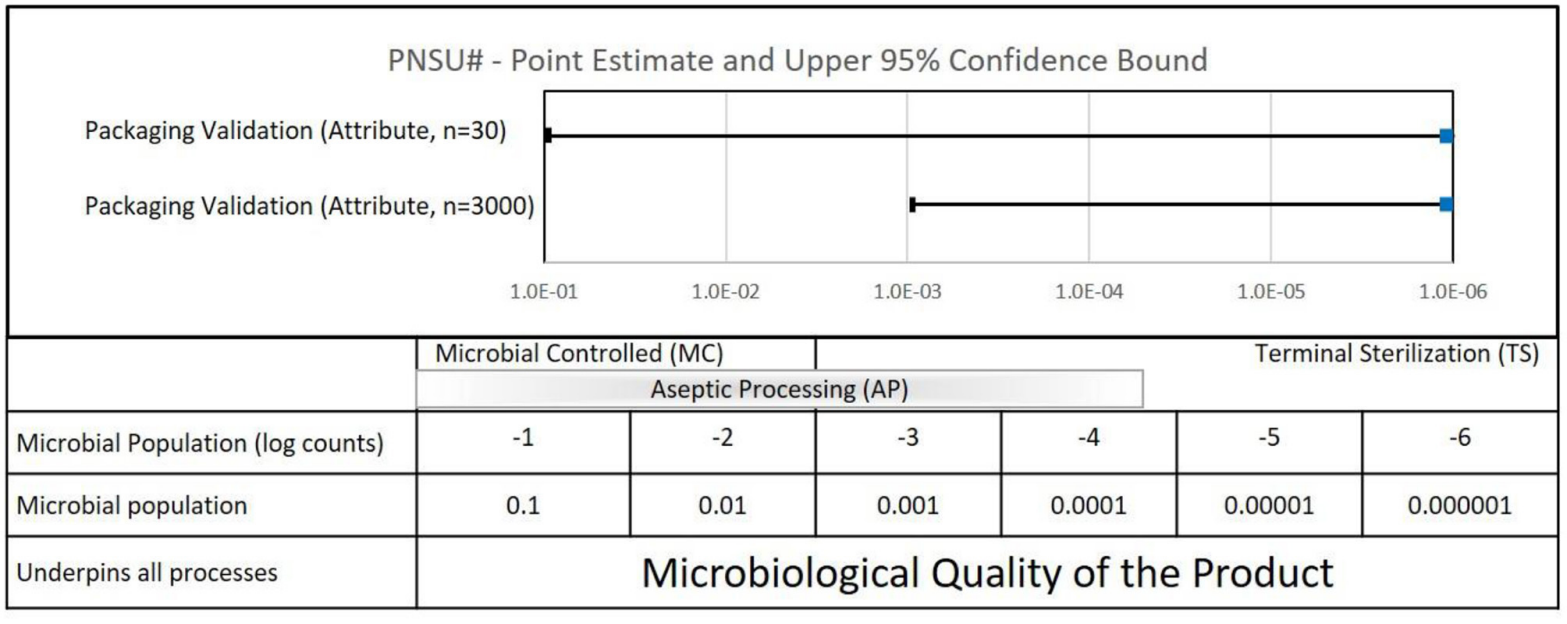
Quantifiable aspects of typical packaging sterile barrier system validations (ISO 11607).

PNSU# results are shown in Figure 6. If the sample size goal was to achieve a PNSU# of <10^−6^, the point estimate and confidence level would be to the left of the dashed line at 10^−6^. In the case of whole package integrity testing performed during a packaging sterile barrier system validation with no failures, the point estimates for both a sample size of 30 and 3,000 are zero, represented as 10^−9^ on the graph. However, the confidence intervals are 10^−1^ and 10^−3^, respectively. Both of these values are significantly >10^−6^, i.e., to the right of the dashed line. To achieve a PNSU# value of 10^−6^ with 95% confidence, some 3,000,000 samples would be required.

What does the calculation of PNSU# tell us? Mostly, it emphasizes to us that to have confidence in a packaging sterile barrier system, it requires more than testing 30 or 3,000 samples for whole package integrity—it requires compliance to the entirety of the ISO 11607 standard. Please note that it does not tell us to increase our testing sample sizes.

#### Aseptic Processing

**Figure 7:**
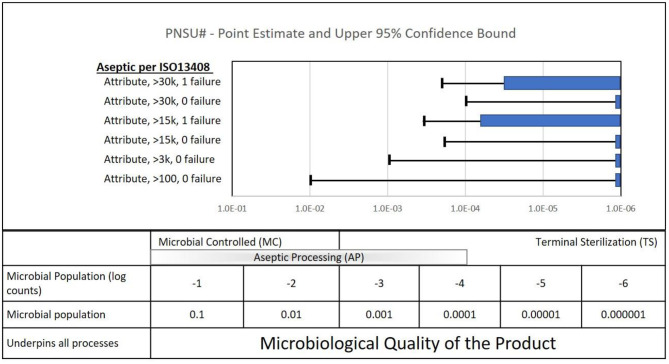
Quantifiable aspects of the sample sizes called out in the ISO standard for aseptic processing (ISO 13408-1).

PNSU# results are shown in Figure 7. Again, if the sample size goal was to achieve a PNSU# of <10^−6^, the point estimate and confidence level 10^−6^ would be to the left of the dashed line at 10^−6^. Process simulation acceptance requirements for four of the six scenarios listed in ISO 13408-1 are zero out of 30,000, 15,000, 3,000 and 100. All four of these results are shown in Figure 7 with point estimates of zero, represented as 10^−6^ on the graph. The confidence interval of these four are 10^−4^, 10^−4^ to 10^−3^, 10^−3^, and 10^−2^, respectively. Again, despite having point estimates of zero, the confidence interval values are significantly greater (a greater mathematical value but not a greater sterility assurance) than 10^−6^. Per the packaging sample size discussion above, to achieve a PNSU# value of 10^−6^ with 95% confidence, some 3,000,000 samples would be required.

Two of the six process simulations requirements permit one failure, out of more than 30,000 and 15,000. These requirements lead to PNSU# point estimates between 10^−5^ and 10^−4^ and confidence intervals between 10^−4^ and 10^−3^.

What does the calculation of PNSU# tell us? Mostly, it again emphasizes to us that to have confidence in the sterility of an aseptically processed product, it requires more than running process simulations required in ISO 13408. It requires compliance to the entirety of the standard. Please note again that it does not tell us to increase our process simulation sample sizes. Aseptic processing with the requirement of ISO 13408 with this range of sample sizes has served patients well for decades. When combined with the entirety of ISO 13408 and other standards, there is high confidence in the manufacture of sterile product. Note: ISO 13408-1 and other industry standards provide for confidence in sterile aseptically processed product through robust characterization and control of the manufacturing environment and equipment; personnel training, gowning, and general health requirements; and manufacturing aseptic technique. Process simulation requirements are provided as a part of the process to qualify the process, with appropriate tests for sterility (2).

#### Terminal Sterilization

**Figure 8:**
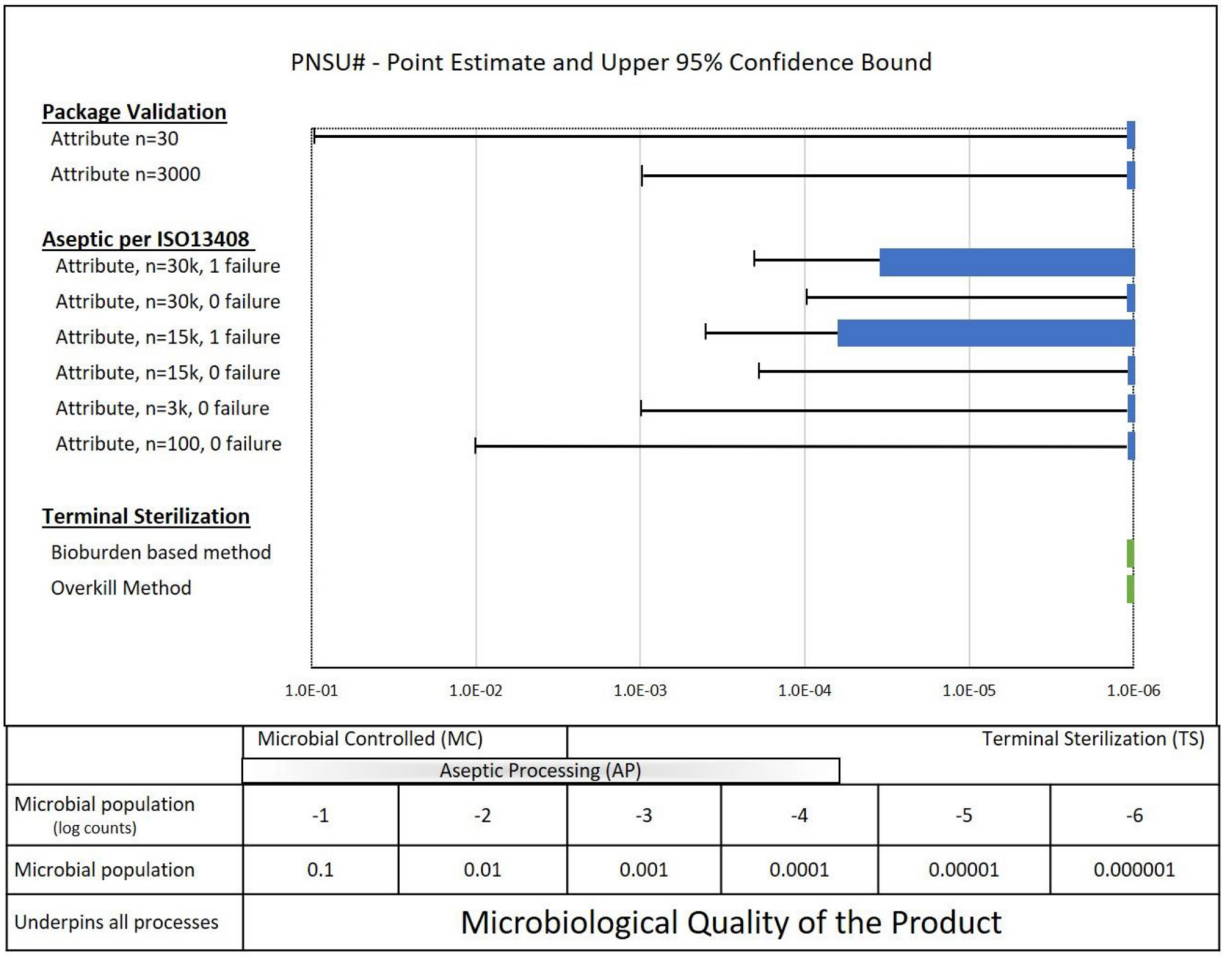
PNSU# values for Terminal Sterilization (ISO 11135 and ISO 11137) along with a summary of packaging results and aseptic processing results.

PNSU# results are shown by the green bars in Figure 8 together with repetition of the results from Figures 6, 7 for packaging validations and aseptic process simulations. As noted above, if the sample size goal was to achieve a PNSU# of <10^−6^, the point estimate and confidence level would be to the left of the dashed line at 10^−6^. This was achieved for the terminal sterilization overkill method and nearly achieved for the terminal sterilization bioburden-based method. These fairly complex variable data parametric PNSU# calculations are based on distributions of bioburden values and modeling of the validation methods in ISO 11135 and ISO 11137. It is interesting to note that finding a bioburden value in Speck et al. (12) for the bioburden-based methods in ISO 11137 to show a confidence interval >10^−6^ was challenging; most bioburden levels resulted in confidence intervals ≤10^−6^ (a lesser mathematical value, not a lesser sterility assurance).

What do these calculations of PNSU# tell us? Since the PNSU# values are less than or close to less than 10^−6^, does it mean that running validations with the sample sizes in ISO 11135 and ISO 11137 standards are sufficient to achieve an SAL of 10^−6^? Absolutely not. To have confidence in the sterility of terminal sterilized product, it requires more than running validations with the sample sizes per the standards—it requires compliance to the entirety of the standards. Despite the reality that running validations with the samples sizes required in ISO 11135 and in the ISO 11137 series of standards has served the industry well for decades, it is mandatory that the entirety of standards be followed to have confidence in the manufacture of sterile product. Note: ISO 11135 and 11137 provide for confidence in terminally sterilized product through robust control of product bioburden through manufacturing and personnel controls and through a rigorous and sequential validation process.

### Summary—Quantifiable Portions of Sterility Assurance Standards—PNSU#

A new PNSU# concept has been developed. A definition has been proposed for conversation with the sterility assurance community. The PNSU# term is not a replacement for either the SAL or PNSU term. It is intended to complement these terms, provide for communication across sectors, and help the industry put sample sizes into perspective without being enamored or frightened by them. It is a proposed new tool in the toolbox of sterility assurance professionals in evaluating sterility assurance challenges facing the industry.

### Summary—New Paradigms and Tools in Sterility Assurance

Terminal sterilization is preferred over aseptic processing when materials of the product are compatible with an SAL of 10^−6^. When, however, it is not possible to achieve an SAL of 10^−6^, aseptic processing has in practice historically been overwhelmingly selected over alternative SALs, mainly attributed to a greater familiarity and understanding of aseptic processing. The new paradigms and tools outlined in this article have the potential to open doors for re-assessing the use of terminal sterilization as SALs >10^−6^, e.g., 10^−4^, and other options for addressing the challenges facing the industry.

## Next Steps

The new paradigms and the tools addressed in this article can be applied to analyze and provide innovative solutions for challenges the healthcare industry is facing. However, the concepts discussed here need to be more widely understood in order to be put into practice. The outcome of applying alternative SALs can then become another option to solving challenging sterility assurance issues. A better understanding of sterility assurance concepts can be gained by the following.

### Education

#### Objectives

- Better understanding of the logic, science and safety of alternative SALs.
- Road mapping how to support alternative SAL solutions to deliver sensitive products.
- Deeper understanding of relative risk of aseptic processing, alternative SALs and various sterility assurance approaches (see Figure 8).

#### Possible Actions

- Kilmer Community Conference or Webinar, sponsored by the Kilmer Collaboration Teams.
- Association for the Advancement of Medical Instrumentation (AAMI)—Webinar or Training Course.
- Parenteral Drug Association (PDA)—Webinar or Training Course.

### Sterilization Standards

#### Objective

- Development and revision of sterilization standards and guidance documents incorporating more information and examples on the application and framework of alternative SALs.

#### Possible Actions

- Aseptic processing standards (Table 2)—include perspective on SALs >10^−6^, e.g., 10^−4^, and references to ISO TS19930 (and ST67) in ISO 13408 when the preference for terminal sterilization over aseptic processing is discussed
- Terminal sterilization Standards (Table 1)—add references to ISO TS19930 (and ST67) and include SALs >10^−6^, e.g., 10^−4^, as examples where appropriate
- Publish end to end sterility assurance framework that will support alternative SALs (AAMI TIR100, in development).

### Publication

#### Objective

- Make available peer reviewed literature on risks associated with use of SALs >10^−6^, e.g., 10^−4^, relative to other options for providing sterile product to patients.

#### Possible Actions

- Publish industry experience with SALs >10^−6^, e.g., 10^−4^, and their associated risk assessments, specifically more details for performing risk analyses for various products and scenarios (e.g., expand on examples from AAMI ST67 and ISO/TS 19930).
- Publish additional practical information on the source of HAIs (e.g., environmental, and personnel) and the impact on patient safety vs. the safety provided by STERILE healthcare products.
- Publish historical data about the safety of SALs >10^−6^, e.g., 10^−4^, that have been used for decades (e.g., non-patient contacting products and products that cannot withstand terminal sterilization to a SAL 10^−6^).

### Microbiological Quality/Microbiological Risk

#### Objective

- Expand the understanding of the Microbiological Quality aspects of healthcare products as it relates to microbiological risk to the patient.

#### Possible Actions

- Promote the understanding that Microbiology Quality as the framework to which all of microbial control, from microbial reduction all the way to terminal sterilization, is measured. It includes a spectrum of methodologies as shown in Figure 8.
- Outline the spectrum of microbiological quality as pertains to the different types of microbiologically controlled product.
- Emphasize in relevant documents that Microbiological Quality is needed to drive the necessary microbiological control or sterility (e.g., 10^−2^ SAL for a low risk product vs. automatically using an SAL of 10^−6^).
- Expand information about the way the microbiological quality is controlled across the spectrum from 10^3^ to 10^−6^.
- Provide information on how multiple processes might achieve the selected level of microbiological quality (e.g., aseptic processing plus a lower level of sterilization to further reduce the bioburden).

## Conclusions

The sterility assurance community needs to have broad discussions about the sterility assurance challenges it is facing. Patients around the world need the innovative medical products being developed, and we need to be responsible in caring for the environment in the process. Recent developments in sterilization standards, ISO/TS 19930 in particular, and in the literature, Speck et al. (12) in particular, provide new paradigms and tools for this discussion. Perhaps it is time that SALs >10^−6^, e.g., 10^−4^ or 10^−5^, be applied as widely and with as much confidence as aseptic processing and that this approach along with bioburden-based sterilization validations be applied to provide terminal sterilization solutions to traditionally aseptically processed product.

## Author Contributions

All authors listed have made a substantial, direct and intellectual contribution to the work, and approved it for publication.

## Conflict of Interest

TB was employed by company BryKor, LLC. The remaining authors declare that the research was conducted in the absence of any commercial or financial relationships that could be construed as a potential conflict of interest.

## Publisher's Note

All claims expressed in this article are solely those of the authors and do not necessarily represent those of their affiliated organizations, or those of the publisher, the editors and the reviewers. Any product that may be evaluated in this article, or claim that may be made by its manufacturer, is not guaranteed or endorsed by the publisher.

## Footnotes

^1^The EPA related pressure on 50% of the medical device sterilization market, ethylene oxide sterilization, is highlighted by the FDA [FDA, Ethylene Oxide Sterilization Facility Updates. https://www.fda.gov/medical-devices/general-hospital-devices-and-supplies/ethylene-oxide-sterilization-facility-updates (accessed December 14, 2020)]. Another 40% of the sterilization market, gamma radiation sterilization, is also under pressure from initiatives to reduce reliance on radioactive materials [https://iarc.fnal.gov/food-and-medical-device-sterilization/ (accessed December 14, 2020)].

^2^ISO 11139:2018. *Sterilization of health care products—Vocabulary of terms used in sterilization and related equipment and process standards*.

^3^The ISO 11139:2018 definition of “terminal sterilization” is process whereby a product is sterilized within its sterile barrier system. The ISO 11139:2018 definition of “sterilization” is validated process used to render product free from viable microorganisms. In practice, EN 556-1 and ANSI/AAMI ST 67 define the required probability of a device having a microorganism following sterilization.

^4^The ISO 11139:2018 definition of “aseptic processing” is handling of sterile product, containers, and/or devices in a controlled environment in which the air supply, materials, equipment, and personnel are regulated to maintain sterility.

^5^ISO 11139:2018 definition of SAL is “probability of a single viable microorganism occurring on an item after sterilization. Note 1 to entry: It is expressed as the negative exponent to the base 10. The requirement of 4.1 EN 556-1:2001 translates into an SAL of 10^−6^. Hence, the probability of a microorganism on one million devices should be less than or equal to one. Increasing sterility assurance translates to smaller numbers, e.g., 10^−7^.

^6^For example drapes and gowns.

^7^ISO 11139:2018. *Sterilization of health care products—Vocabulary of terms used in sterilization and related equipment and process standards*.

^8^https://www.ema.europa.eu/en/documents/scientific-guideline/guideline-sterilisation-medicinal-product-active-substance-excipient-primary-container_en.pdf (accessed December 14, 2020).

^9^Largely taken from https://www.iest.org/Standards-RPs/ISO-Standards/ISO-14644-Series (accessed September 22, 2020).

^10^^*^WHO, Healthcare-associated infections FACT SHEET. http://www.who.int/gpsc/country_work/gpsc_ccisc_fact_sheet_en.pdf (accessed February 27, 2019).

^11^ISO 11139:2018, ISO 11139:2018, *Sterilization of health care products—Vocabulary of terms used in sterilization and related equipment and process standards*.

^12^The definition comes from TR 1:2007, Validation of Moist Heat Sterilization Processes: Cycle Design, Development, Qualification, and Ongoing Control. Note: The mission of the PDA is advance pharmaceutical/biopharmaceutical manufacturing science and regulation so members can better serve patients.

^13^ISO/TS 19930 clause 4.5.
